# Determinants of COVID-19 vaccine behaviour intentions among the youth in Kenya: a cross-sectional study

**DOI:** 10.1186/s13690-022-00904-4

**Published:** 2022-06-23

**Authors:** Joachim O. Osur, Rehema Chengo, Evelyne Muinga, Jackline Kemboi, Myriam Sidibe, Maggie Rarieya

**Affiliations:** 1 Department of Rehabilitative Medicine, School of medical Sciences, Amref International University, P.O. Box 27691-00506, Nairobi, Kenya; 2 Department of Technical Directorate Unit, Amref Health Africa Headquarters, P.O. Box 27691-00506, Nairobi, Kenya; 3 London School of Hygiene and Tropical Medicine and Brands on a Mission, London, UK; 4 The National Business compact on Corona, Nairobi, Kenya

**Keywords:** Vaccine hesitancy, Safety, Effectivenes

## Abstract

**Introduction:**

COVID-19 has become a public health concern globally with increased numbers of cases of the disease and deaths reported daily. The key strategy for the prevention of COVID-19 disease is to enhance mass COVID-19 vaccination. However, mass vaccination faces challenges of hesitation to acceptance of the vaccine in the community. The youth may not be among the vulnerable population to severe COVID-19 disease but are highly susceptible to contracting the virus and spreading it. The aim of the study was to assess COVID-19 vaccine youth behavior intentions and their determinants in Kenya.

**Methods:**

The study used a mixed method design, employing a cross-sectional survey and focused group discussions across 47 counties in Urban, Peri-Urban and Rural settings of Kenya. The interviewees were youths aged 18–35, registered in online platforms/peer groups that included Shujaaz, Brck Moja, Aiffluence, Y Act and Heroes for Change. Quantitative data was collected using Google forms. A total of 665 interviews were conducted. Quantitative data was analysed using STATA version 16. In this paper we report quantitative study findings.

**Results:**

The findings of the study indicated that only 42% of the youth were ready to be vaccinated, with 52% adopting a wait and see approach to what happens to those who had received the vaccine and 6% totally unwilling to be vaccinated. The determinants of these behavior intentions included: the perceived adverse effects of the vaccine on health, inadequate information about the COVID-19 vaccine, conflicting information about COVID-19 vaccine from the social media, religious implications of the vaccine, impact of education level on understanding of the vaccine, perceived risk of contracting the COVID-19 disease, efficacy of the COVID-19 vaccine, COVID-19 affecting women than men and trust in the ministry of health to lead COVID-19 interventions. Significantly it was found that hesitancy is higher among females, protestants and those with post-secondary education. Lack of information and concerns around vaccine safety and effectiveness were main cause of COVID-19 vaccine hesitancy. Social media was the major source of information contributing to hesitancy. Other contributors to hesitancy included low trust in the MoH and belief that mass vaccination is not helpful.

**Conclusion:**

Vaccine hesitancy remains high among the youth but the causes of it are modifiable and health systems need to have evidence based engagements with the youth to reduce vaccine hesitancy.

## Background

Coronavirus disease 2019 (COVID-19) is an infectious viral disease spread through respiratory droplets. COVID-19 spreads through aerosols when people are in close contacts such as in crowded places and places with poor ventilation [1]. The rapid spread of COVID-19 disease has caused significant strain to health systems, led to deaths and ill health, and raved economies globally [2–5].

The confirmed cases of COVID-19 have risen globally with over 96,658,420 cases and 2, 092,062 deaths reported as of 23rd January 2021. In Africa approximately 2, 395,260 cases and 55,644 deaths were confirmed, while in Kenya 99,444 cases and 1, 736 deaths have been confirmed [1].

Several measures have been put in place to contain the disease including social distancing, wearing of masks and washing of hands and use of disinfectants [6]. Additionally, it is anticipated that vaccination of the population will help to prevent transmission of the disease. The world is racing to get an effective and safe vaccine with the aim of vaccinating a critical mass of the world population to achieve herd immunity [7].

The greatest concern right now is that populations may not fully accept the COVID-19 vaccine. Increasing vaccine hesitancy have been noted globally [1, 4, 8–10]. Vaccine resistance and hesitancy can be caused by numerous factors including misinformation, lack of knowledge on vaccines, myths, social norms, traditional and religious beliefs, misconceptions, incitement from peers and politicians [5, 11].

Additionally, safety and efficacy concerns of the vaccine have strong relationship with individual’s willingness to be vaccinated and can affect uptake and increase COVID-19 vaccine hesitancy [12]. Also, the belief that the vaccine can cause adverse side effects results in lowered confidence in the COVID-19 vaccine and reduce uptake of the vaccine [6, 13, 14].

An individual’s motivation for vaccination with the COVID-19 vaccine is influenced by their confidence in the vaccine, feelings, and how they perceive themselves to be at risk of contracting COVID-19 disease. If people think that they are at low risk of contracting the disease, they are likely to be less motivated to vaccinate with the COVID-19 vaccine. If they feel the vaccine is not beneficial, they are less likely to be motivated to receive the vaccine [6]. Lack of knowledge of the risks of not being vaccinated and the benefits of the COVID-19 vaccine is also a contributing factor to the lack of intentions to receive the vaccine once it is made available to the populace [15].

Research shows that individuals who don’t perceive to be at risk of infections are less likely to be vaccinated. A review of the study of H1N1 revealed that one of the major determinants of intentions to accept a vaccine is not being bothered about the influenza vaccine. This is likely to lower vaccine demand among the youths, hence lower uptake of the vaccine. Also, when a group of people believe that a disease is mild, the intentions to receive the vaccines are lowered [16].

Moreover, conspiracy theories and vaccine opposition are increasing across the world as roll out of the COVID-19 vaccine is being undertaken [17, 18]. In Nigeria and Pakistan, research reveals that waves of misinformation about polio vaccine which has been circulating in the social media, as well as religious extremism have led to a decline in uptake of the polio vaccine, hence increased cases of polio in the respective countries [19]. Such misinformation can easily affect COVID-19 vaccination.

The severity of COVID-19 disease occurs in people with advanced age as well as those with diabetes, hypertension, heart disease, and cancer compared to young people [9, 20]. However, the youth may be more susceptible to contracting COVID-19 disease than older people, because of their low perceived risks, which may result in transmitting the disease to their loved ones. Also, health workers who are exposed to infection by the nature of their work, do suffer a high burden of disease and some are youth. In a phased-off COVID-19 vaccine approach, therefore, health workers, the elderly, and those with pre-existing health conditions are prioritized for COVID-19 vaccination. It is however important to note that acceptance of the vaccine by these groups is not necessarily given. Vaccine hesitancy is known to be a household and community behavior rather than just individual health-seeking behavior. The WHO Technical Advisory Group on Behavioral Insights and Sciences for Health has prioritized social influence as an important factor in determining COVID-19 vaccine uptake [6]. As such, health workers, as well as the aged and people living with pre-existing health conditions, are likely to be influenced by and also influence the youth in their behavior intentions towards the COVID-19 vaccine.

The role of social media is identified as an important factor in social influence. The youth have access to social media and are likely to interact much more with negative messages and conspiracy theories on COVID-19 vaccination which may lead to vaccine hesitancy [12, 21, 22]. They are likely to not only develop vaccine hesitancy through the influence of social media but also act as agents of vaccine hesitancy in their families and communities [23]. Other studies have similarly found that adults are influenced by their social contacts in accepting vaccination and the social circle may involve the youth that they associate with [17]. However, there are no studies done to establish the behaviour intentions of youth on the COVID-19 vaccine and the determinants of the intentions [22]. Studying COVID-19 behavior intentions and their determinants among the youth is an important entry point to understanding possible vaccine hesitancy issues in families and communities and help in designing behaviour change communication for increasing vaccine uptake among all members of the community.

## Methods

### Aim of the study

This study aimed to assess COVID-19 vaccine youth behavior intentions and their determinants in Kenya. Specifically, the study sought to determine COVID-19 vaccine behavior intentions and establish the determinants of the COVID vaccine hesitancy on COVID-19 vaccine among the youth in Kenya.

### Study design

This study utilized mixed-method study using a cross-sectional survey and focused group discussions approaches. Determinants of behavior intentions on the COVID-19 vaccine among the youths were measured quantitatively by cross-sectional survey while focused group discussions was used to establish the reasons behind the variables measured. This is a report of the findings of the cross-sectional survey.

### Study setting and target population

The study was conducted in 47 counties of Kenya covered by the youth online platforms from where participants were drawn. The target population were youths aged 18–35 years as per the 2010 Kenyan constitution definition of youth. The online platforms had a list of actively registered users, which made it easy for them to be sampled. The NBCC a partner in the study put together social businesses that targeted the youth and had identified the platforms that they had registered including Heroes 4Change, Aifluence, MojaWifi, Brick Moja, Y act, and Shujaaz Inc. Youth who had registered and were active in the online platforms were eligible to take part in the study. The study excluded inactive registered users as well as unregistered youths that were not on the online platforms. In addition, youths who refused to give consent to participate in the study were excluded from the study.

### Sampling techniques and sample size determination

#### Sampling technique

The number of active youths registered on the online platforms formed the sampling frame for the study. Convenient sampling was used to select the online platforms because they contained the target population and the population who could access the online surveys. The youth were selected from each online platform using random sampling to give each member an opportunity to participate and prevent selection bias. Sample distribution across the platforms was based on proportion to size of the number of registered youths in each of platforms.

#### Sample size determination

There were 53,289 active registered online platform users in the areas of study, that is, Shujaaz Inc. 40,000; Brck Moja 6000; Y act 3000; Heroes4Change 419 and AIfluence 3870. Krejcie and Morgan sampling formula (1970) was used to determine the sample size of the youths to be surveyed in each platform because the number of registered youths on the online platform is known.

$$\mathrm{s}={\upchi}^2\mathrm{NP}\left(1-\mathrm{P}\right)\div {\mathrm{d}}^2\left(\mathrm{N}-1\right)+{\upchi}^2\mathrm{P}\left(1-\mathrm{P}\right)$$

Where,

s = required sample size.

X^2^ = the table value of chi-square for 1 degree of freedom at the desired confidence level *p* = 0.05 (3.841).

*N* = the population size (53289).

*d* = with the degree of accuracy expressed as proportion (0.05).

10% of the sample size was added to the original sample to account for non-response or attrition rates.

A total of 665 youths were drawn from the five platforms. The sample was distributed proportionately according to the number of registered members per platform. Random sampling was used to select youths to be surveyed from each platform..

### Development of research instruments

The cross-sectional survey structured questionnaires were adapted from the Health Belief Model (HBM), Theory of Planned Behavior (TPB), and WHO SAGE matrix [1]. The questions focused on examining the determinants of behaviors on the COVID-19 vaccine and behavior intentions on accepting or hesitating to take up the vaccine when available to the youths.

### Data collection techniques

The quantitative data collection was administered using google forms. The questionnaires were designed on Google forms, and then the database secured using a strong password. An automatic web URL link was generated and the link was shared to the research assistants via the email. The first section of the form contained the consent form, of which the participants were only allowed to participate once they consented.

### Data management and analysis

The quantitative data was transferred into STATA version 16, cleaned, and coded for analysis. The results of the quantitative variables are summarized into descriptive statistics. The univariate analysis was used to analyze continuous variables while chi-square test was used to analyze categorical variables to establish the determinants of behavior intention amongst the youths on the COVID-19 vaccine. Structural equation modeling was employed to establish the significant association of HBM and TPB that might have a considerable effect on youth’s intention to accept the COVID-19 vaccine.

## Results

### Demographics data

The sample included 665 youths (60.3% male) and (30.7% females) among which 45.4% were from urban counties, 26.4 and 28.3% are from peri-urban and rural counties respectively.^1^ 55.3% of the youth interviewed were aged 18 to 23 years, 41.0% are from urban counties, 31.3% are from rural counties and minority 27.7% are from peri urban counties. Among the youths surveyed, 48.9% of them were protestants and lived in urban counties. The education level of the youths interviewed were college/university (62.7%) where 64.2% of them came from urban counties.

### Behavior intentions

**Fig. 1:**
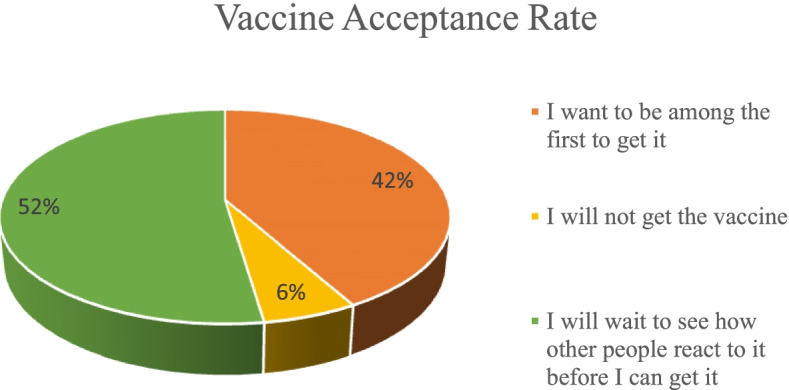
Vaccine acceptance rate

52.0% of the youths surveyed reported that they would wait and see how vaccinated people reacted to the COVID-19 vaccine before they could get it, and 42.0% reported that they would be among the first people to be vaccinated *(*Fig. 1). In contrast, 6.0% of the youths stated they would not take the vaccine. Disagregating by education level, the study revealed a significant relationship between educational level and COVID-19 vaccine uptake with *p*-value < 0.05. Further analysis showed a moderate association between education level and COVID-19 vaccine uptake (Cramer’s v = 0.1).

Similarily, the study revealed that majority (60.9%) of the females would wait and see how other people would react to the vaccine before they could get it while 47.4% of male participants would be among the first people to get the vaccine. Statistically there was a significant difference between gender and uptake of COVID-19 vaccine with *p*-value < 0.05. Further test showed a moderate association between gender and uptake of COVID-19 vaccine (Cramer’s V = 0.1). The study also revealed a significant association between religion and vaccine uptake (*P*-value < 0.05). See Table 1.

**Table 1:** Socio demographic factors by intention to get CoVID-19 vaccine

Variable	I want to be among the first people to get it n(%)	I will not get the vaccine n (%)	I will wait to see how other people react to it before I can get it n (%)	*P*-Value (Cramer’s V)
**Age**				
18 to 23 years	150(40.8)	15(4.1)	203(55.2)	
24 to 29 years	110(44.2)	22(8.84)	117(46.99)	0.06
30–35 years	18(37.5)	2(4.2)	28(58.3)	
**Gender**				
Female	87(33.7)	14(5.4)	157(60.9)	0.004(0.1)
Male	190(47.4)	24(6.0)	187(46.6)	
Prefer not to say	1(16.7)	1(16.7)	4(66.7)	
**Religion**				
Catholic	148(51.2)	10(3.5)	131(45.3)	
Islam	4(26.7)	1(6.7)	10(66.7)	0.001(0.1)
No Religion	2(66.7)	0(0.00)	1(33.3)	
Protestant	126(34.6)	28(7.8)	206(57.5)	
**Education Level**				
College/university	149(35.75)	29(6.94)	239(57.42)	
Primary	8(42.1)	1(5.26)	10(52.63)	0.001(0.1172)
Secondary	121(53.1)	9(3.95)	99(42.98)	
**County Category**				
Peri Urban Counties	84(46.7)	8(4.4)	88(48.9)	0.1
Rural Counties	85(45.7)	10(5.4)	91(48.9)	
Urban Counties	109(36.5)	21(7.0)	169(56.5)	

**Fig. 2:**
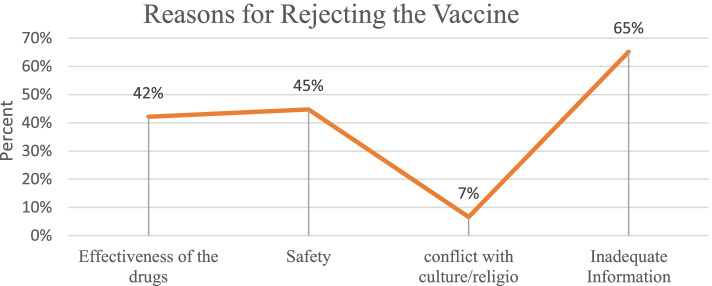
Reason for Rejecting COVID-19 Vaccine

65.0% of the youths correlated the rejection of COVID-19 vaccine to inadequate information, effectiveness of the drug and safety was 42.0 and 45.0% respectively. Only 7.0% of the youths reported that their reason for rejecting the COVID-19 vaccine was due to conflict with culture or religion *see* Fig. 2*.*

The results further revealed that there was a widening gap in access to the COVID vaccine among females and males, sometimes men tending to access the vaccine more than the women.

Multinomial logistic regression was also carried out to determine significant predictors in the uptake of the COVID-19 vaccine,^2^ see Table 2. The results indicated that male predictor were 0.6 less likely to wait to see how other people reacted to the vaccine before they could get it and were more likely to be among the first people to get the vaccine compared to the females . The test was statistically significant with *p*-value < 0.05.

**Table 2:** Associated factors of uptake of COVID-19 using a multivariable multinomial regression

Variable	I will not get the vaccine n (%)			I will wait to see how other people react to it before I can get it n (%)		
	Coefficient	Standard error	***P***-value	Coefficient	Standard error	***P***-value
**Gender (reference Predictor-Female**						
Male	−0.1600	0.3655	0.662	−0.5538	0.1730	0.001
Prefer not to say	1.5445	1.4650	0.292	0.5979	1.1355	0.599
**Religion (reference predictor- Catholic)**						
Islam	1.3392	1.1772	0.253	1.0352	0.6138	0.092
No Religion	−10.6582	489.341	0.983	−0.6089	1.2348	0.622
Protestant	1.1485	30.3905	0.003	0.5853	0.1689	0.001
**Education Level** (**Reference Predictor-College/university)**						
Secondary	−0.9131	0.4046	0.670	−0.6405	0.1754	0.000
Primary	−0.473	1.1047	0.024	−0.1769	0.4990	0.723

Similarily, the youths with primary level of education were 0.6 more likely to get the vaccine and more likely to be among the first people to get the vaccine compared to the respondents who had tertiary/University level of education and the test was statistically significant with *p*-value < 0.05. In addition, the respondents who had secondary level of education were less likely to wait and see how people reacted to the vaccine before they could get it. However,theywere more likely to be among the first people to get the vaccine compared to the respondents who had tertiary/University level of education and this was statistically significant at *p*-value < 0.05.

On the other hand, protestants were 1.1 less likely to get vaccinated and more likely to wait to see how other people reacted to the COVID-19 vaccine before they could get it. The test was statistically significant with *p*-value < 0.05. In addition, they were also 0.6 less likely to be among the first people to get the vaccine compared to Catholic. The test was statistically significant with *p*-value < 0.05.

### Contextual influences – communication and social media, gender and socio-cultural

**Fig. 3:**
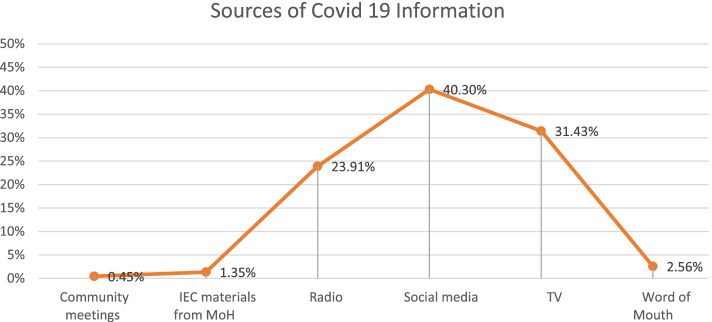
Sources of information on COVID-19 vaccination that you and your community come across

The most common source of information about COVID-19 vaccines was reliance on social media 40.30%. This was followed by TV programs and radio, 31.43 and 23.9% respectively, word of mouth was 2.6%, while IEC material from MOH was the main source of information for 1.4% of the youth. See Fig. 3.

Chi square test was used to determine the relationship between socio-demographic factors and the main source of COVID-19 information. There was an association between gender, educational level and county of residence and the main source of information with *p*-value < 0.05. Social media platforms were the main source of information about COVID-19 among the female youths, respondents residing in urban counties and respondents with college/university educational levels. The male respondents preferred radio as the source of COVID 19 information compared to female. On the hand, the female respondents preferred TV compared to male respondents*.*

Multinomial logistic regression was also carried out to determine significant predictors in gender, religion, level of education and county of residence. In gender, the ‘Male’ predictor was positive and significant *P*-value < 0.05. This predictor revealed that the male youths were more likely to access information on COVID-19 vaccine on radio and less likely on social media as compared to female.

Analysing education level, both primary and secondary predictors were positive and significant *P*-value < 0.05. The positive slope suggests that the youth participants with primary and secondary level of education are more likely to access information on COVID-19 on Radio and less likely on social media as compared to participants with tertiary/ University level of education. Comparing the accessibility of COVID-19 information on TV and social media, participants with secondary level of education were more likely to access information on COVID-19 on TV and less likely on social media.

Looking at youth’s place of resindence, ‘urban category’ predictor was negative and significant with *P*-value< 0.05. The negative slope indicates that participants from urban counties were less likely to access information on COVID-19 on Radio and more likely to access the same information on social media as compared with participants from rural counties.

60.3% of the youths felt that the information on COVID-19 was being shared openly, a chi square test revealed that there a relationship between the uptake of COVID-19 vaccine and the feeling that the information on vaccine was being shared openly. The test was statistically significant with *P*-value < 0.05.

Despite the fact that majority (60.3%) of the respondents believed that the information was being shared openly the youths believed that this information was not sufficient.. Furthermore, 55.3% of the youths had heard the information on social media that would make them reconsider the uptake of the COVID-19 vaccine and the relationship with uptake of the COVID-19 vaccine was statistically significant *P*-value < 0.05.

Majority of the youths surveyed thought that political leader’s sentiments on COVID-19 vaccine could trigger doubts on the vaccine amongst the youths/ the community, and this factor showeda statistical significant associationwith the uptake of COVID-19 Vaccine (Chi square *P* Value < 0.05 at 95% C.I). There was no relationship between the uptake of vaccine and culture.

Multinomial logistic regression was carried out to determine significant predictors in the uptake of COVID-19 vaccine.^3^ The predictor of whether the youths felt that COVID-19 vaccine information was being openly shared was negative and significant at *P*-value < 0.05. This indicated that the participants who felt that the information on the COVID-19 vaccine was being shared openly weremore likely to get vaccinated and more likely to be the first people to get vaccinated as compared to participants who felt that information on COVID-19 vaccine was not being shared openly. They were also less likely to wait and see how people reacted to the vaccine was statistically significant at *P*-value < 0.05.

Looking at ‘Have you heard/ read in the media/ on social media any information that would make you reconsider not to take COVID-19 vaccine’ and the uptake of COVID-19 vaccine, this predictor was positive and significant at *P*-value < 0.05. This indicated that participants who had heard/ read in the social media any information that would make them reconsider not to take COVID-19 vaccine were more likely to wait to see how other people reacted to the COVID-19 vaccine before they could get it and less likely to be among the first people to get vaccinated as compared to those who had not heard/ read in the media/ on social media any information that would make them reconsider not to take COVID-19 vaccine. Table 3.

**Table 3:** Contextual Influences by intention to get COVID-19 vaccine

Variable	I want to be among the first people to get it n(%)	I will not get the vaccine n (%)	I will wait to see how other people react to it before I can get it n (%)	*P*-Value (Crammers V)
Do you feel that information on COVID 19 vaccine is being openly shared?				
No	57(8.57)	30(4.51)	176(26.47)	< 0.000(0.3526)
Yes	221(33.23)	9(1.35)	172(25.86)	
Have you heard/ read in the media/ on social media any information that would make you reconsider not to take COVID-19 vaccine?				
No	144(21.65)	18(2.71)	135(20.30)	0.005(0.1263)
Yes	134(20.15)	21(3.16)	213(32.03)	
Do you think political leader’s sentiments on COVID-19 vaccine can trigger doubts on the vaccine amongst the youths/ the community?				
No	82(12.33)	5(0.75)	63(9.47)	0.001(0.1437)
Yes	196(29.47)	34(5.11)	285(42.86)	
Do you know people in your community who oppose COVID-19 vaccine on cultural grounds?				
No	193(29.02)	29(4.36)	269(40.45)	
Yes	85(12.78)	10(1.50)	79(11.88)	0.083

**Fig. 4:**
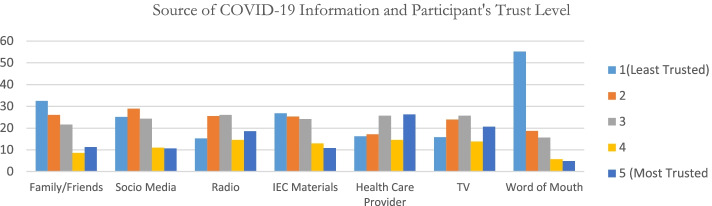
Degree of trust on Sources of information on COVID-19 vaccine

Sharing of relevant vaccine information is key, and the youths surveyed reported that the most trusted source of information was health care providers 26.32% followed by TV and Radio at 20.66 and 18.59% respectively and the least trusted source of information was word of mouth 55.19%. See Fig. 4.

**Fig. 5:**
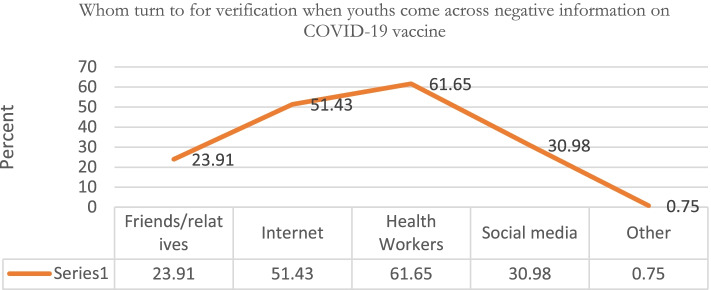
Whom turn to for verification when youths come across negative information on COVID-19 vaccine

Similarly,61.65% of the youth would turn to health care provides for verification when they came across negative information on COVID-19 vaccine as illustrated in Fig. 5.

### Individual and group influences

The study revealed that 30.73% of the youths were influenced by their parents or the vulnerable groups not to receive the vaccine at an average extent.

64.21% of the youths surveyed believed that once they were vaccinated with the COVID-19 vaccine, other people in the community would be protected as well. The association of the youths’ believe that once they are vaccinated the rest of the people in the community will be protected with the uptake of COVID-19 vaccine was statistically significant (*P*-value < 0.05). The study further showed that 71.72% of the youth support mass vaccination, this was also statistically significant *P*-value < 0.05 and the association was moderate. See Table 4.

**Table 4:** Group influences by intention to get COVID-19 vaccine

Variable	I want to be among the first people to get it n(%)	I will not get the vaccine n (%)	I will wait to see how other people react to it before I can get it n (%)	*P*-Value (Crammers V)
Do you believe that once you are vaccinated with COVID-19 vaccine, other people in the community will be protected as well?				
No	66(9.92)	28(4.21)	144(21.65)	*P* < 0.000(0.2581)
Yes	212(31.88)	11(1.65)	204(30.68)	
Do you support mass vaccination (vaccinating everyone) with COVID-19 vaccine?				
No	27(4.06)	32(4.81)	129(19.40)	< 0.001(0.4179)
Yes	251(37.74)	7(1.05)	219(32.93)	

Multinomial logistic regression was carried out to determine youths uptake of COVID-19vaccine, the study revealed that the youths believed that once they were vaccinated with COVID-19 vaccine,^4^ other people in the community would be protected as well’ are 1.4 times more likely to get vaccinated and 0.6 times less likely to wait and see how people reacted to the vaccine before theycould get it as compared to those who did not believe that once they were vaccinated with COVID-19 vaccine, other people in the community would be protected as well. This was statistically significant at *P*-value < 0.05.

In addition the youths who support mass vaccination (vaccinating everyone) with COVID-19 vaccine were more likely to be among the first people who were less likely to wait and see how people reacted to the vaccine before they could get the vaccine compared to those who did not support mass vaccination (vaccinating everyone) with COVID-19 vaccine. This was statistically significant with *P*-value< 0.05 See Table 5.

**Table 5:** Multinomial logistic regression on Individual and Group Influences and vaccine uptake

Variable	I will not get the vaccine n (%)			I will wait to see how other people react to it before I can get it n (%)		
	Coefficient	Standard error	***P***-value	Coefficient	Standard error	***P***-value
Do you believe that once you are vaccinated with COVID-19 vaccine, other people in the community will be protected as well?						
**Reference Predictor-No**						
**Yes**	−1.4466	0.4085	0.000**	−0.5585	0.1882	0.003
Do you support mass vaccination (vaccinating everyone) with COVID-19 vaccine?						
**Reference Predictor-No**						
**Yes**	−3.4201	0.4728	0.000**	−1.5765	0.2347	0.000

### Vaccine safety and vaccination specific determinants

60.0% of the youths surveyed believed that their Country was not able to manage risks associated with COVID-19 vaccine side effects; chi square test was carried out to determine if this relationship with uptake of COVID-19 vaccine was significant, and a *P* Value < 0.05 was obtained. This implied that there was a relationship between country’s readiness to manage the risk associated with COVID-19 vaccine and the uptake of the vaccine was statistically significant. Moreover, 35.7%of the youths were somewhat confident on the safety of COVID-19 vaccine and this relationship with the uptake of vaccine was found to be statistically significant with *P* Value < 0.05. 29.8% were concerned that they could develop serious side effects from the COVID-19 vaccine, the relationship with uptake of COVID-19 vaccine was statistically significant with *P* Value < 0.05. On the other hand, 78.7% of the youths trusted the health system to deliver COVID-19 vaccine to their communities and somewhat agreed that the benefits of being vaccinated with COVID-19 vaccine exceeded the risks of not being vaccinated. These relationships were statistically significant with *P*-value < 0.05.

Multinomial logistic regression was carried out to determine significant predictors in the uptake of COVID 19 vaccine.^5^ The predictor ‘Can the health system be trusted to deliver COVID-19 vaccine to your communities?’ was negative and significant at *P*-value< 0.05. The negative slope indicates that participants who trust that the health system could deliver COVID-19 vaccine to their communities were 0.6 times more likely to get vaccinated and 0.5 times less likely to wait and see how people could react to the vaccine before they could get it as compared to those who trusted the health system to deliver COVID-19 vaccine to their communities.

The predictor ‘How confident are you in the safety of COVID-19 vaccine?‘is positive and significant *P*-value < 0.05.The positive slope indicated that participants who were not confident in the safety of COVID-19 vaccine were 3.4 times more likely not to get vaccinated and 2.4 times more likely to wait and see how people reacted to the vaccine before they could get it as compared to those who were confident in the safety of COVID-19 vaccine.

The predictor ‘How much do you agree that benefits of being vaccinated with COVID-19 vaccine exceeds the risks of not being vaccinated?’ was positive and significant at *P*-value< 0.05. The positive slope indicated that participants who strongly disagreed that benefits of being vaccinated with COVID-19 vaccine exceeded the risks of not being vaccinated were 1.7 times more likely not to get vaccinated and less likely to be among the first people to get vaccinated as compared to those who strongly agree that benefits of being vaccinated with COVID-19 vaccine exceeds the risks of not being vaccinated. See Table 6.

**Table 6:** Multinomial logistic regression on Vaccine safety and vaccination specific Determinants and vaccine uptake

Variable	I will not get the vaccine n (%)			I will wait to see how other people react to it before I can get it n (%)		
	Coefficient	Standard error	***P***-value	Coefficient	Standard error	***P***-value
Do you feel our country is able to manage risks associated with COVID-19 vaccine side effects?						
**Reference Predictor-No**						
**Yes**	−0.4284	0.5174	0.408	−0.3836	0.2034	0.059
Can the health system be trusted to deliver COVID-19 vaccine to your communities?						
**Reference Predictor-No**						
**Yes**	−1.4831	0.4866	0.002***	−0.5388	0.2745	0.050
How confident are you in the safety of COVID-19 vaccine?						
**Reference Predictor- Confident**						
**Very confident**	−0.3256	0.8549	0.703	−0.7059	0.2872	0.014
**Somehow confident**	−0.2544	0.9400	0.787	1.3098	0.2345	0.000
**Not confident**	3.4989	0.7926	0.000***	2.4461	0.4456	0.000
**Not confident at all**	18.0808	585.3956	0.975	15.6067	585.3953	0.979
**Don’t know**	3.5272	1.0341	0.001	2.1140	0.6583	0.001
How much do you agree that benefits of being vaccinated with COVID-19 vaccine exceeds the risks of not being vaccinated?						
**Strongly Agree**	0.7667	0.9146	0.402	−0.5532	0.2906	0.057
**Neutral**	0.3004	0.8795	0.733	0.2830	0.302	0.302
**Disagree**	1.6277	0.8706	0.062	0.4772	0.3093	0.123
**Strongly Disagree**	1.7319	0.8850	0.050***	−0.1375	0.3457	0.691

### Vaccine knowledge questions

86.17% of the respondents did not know what the COVID-19 vaccine contained and 46.92% of them did not know the effectiveness of COVID-19 vaccines in preventing infection as shown in Table 7.

**Table 7:** COVID 19 Vaccine knowledge

Variable	Frequency	Percent(%)
Do you know what the COVID-19 vaccine contains?		
No	573	86.17
Yes	92	13.1.83
Do you know the effectiveness of COVID-19 vaccines in preventing infection?		
a. Is quite high in vaccines that have been approved	226	33.98
b. It will be known once the COVID-19 vaccines are in use for a while	127	19.10
c. I do not know	312	46.92

## Discussions

The study findings indicated that a relative high proportion of the youths had intentions of being vaccinated with COVID-19 vaccine, while more than half of the youths had adopted “The wait and see” approach on the COVID-19 vaccine, that is effects of the COVID-19 vaccine on people who had been vaccinated. These results were similar with findings from a study done in Portugal, which indicated that more than half of the community members would wait and see how other people reacted to the vaccine majority being young people [24]. However, the proportion of youths unwilling to be vaccinated with COVID-19 vaccine indicated vaccine hesitancy that needs to be addressed. According to a study done in India among the youths in college, similar findings were observed where majority of the youths had positive intentions of being vaccinated [15]. These results were contrary to findings of a study done in Netherlands which indicated that high percentage (78%) of youths were willing to be vaccinated [25].

From the study, education level of the youths had a significant association with the uptake of COVID-19 vaccine. Youths with higher education level were less likely to take the vaccine compared to youths with lower education levels. Contrary to a study done by Soares et al., (2021) which found that youths with higher levels of education were more likely to take the COVID-19 vaccine. This study’s findings contrasted a study done in UK which revealed that people with lower education level were less likely to take up the vaccine compared with those with high education level [13]. There was a significant difference between gender and uptake of COVID-19 vaccine. Female youths were more hesitant to uptake of COVID-19 vaccine compared to male youths, most of them adopting ‘Wait and see approach’. The results were similar to findings of a study done by Fazel et al., 2021 which showed that females were less likely to receive the COVID-19 vaccine. Contrary, a study by Jain et al., 2021 indicated that gender did not have any association with uptake of COVID-19 vaccine among the youths [26–28].

The COVID-19 vaccine hesitancy among the youths was attributed to lack of adequate information about the vaccine, the effectiveness of the COVID-19 vaccine, safety of the COVID-19 vaccine, and cultural and religious factors. Evidence from a study done in Portugal indicated that safety, effectiveness, and lack of sufficient information about the COVID-19 were major causes of COVID-19 vaccine hesitancy among the youths [24]. Similary, a study done in Pakistan and European countries showed that COVID-19 vaccine safety was associated with the low uptake of the vaccine among their population [29, 30]. Similarly, in a study done in Brazil, South Africa, and UK, perceived safety and efficacy of the COVID-19 vaccine would result in vaccine hesitancy [31].

In this study, religion and culture was mentioned to influence the uptake of the COVID-19 vaccine to a small extent among the youths. Similary in Indonesia, religion was found to cause safety concerns of the COVID-19 vaccine among the Muslims, thus lowered COVID-19 vaccine acceptability [16]. Contrary to the findings of this research, a study done in Italy and Indonesia, found out that religion had no association with uptake of COVID-19 vaccine among the youths [32, 33].

Sharing of right information about the vaccine is a key determinant of COVID-19 vaccine acceptance especially among the youths. The findings showed that majority of the youths trusted information about COVID-19 vaccine they received from health care providers more than other sources. They also reported to trust information on COVID-19 vaccine they received from the TVs and radios. The youths were found to rely more on social media to obtain information about COVID-19 vaccine. These findings were similar to a study done in Pakistan, which indicated that most youths relied on printed or social media for COVID-19 vaccine information [30]. The youths reported that the information they received from social media would make them reconsider not to take the COVID-19 vaccine. Thus, availability of misinformation of COVID-19 vaccine from the social media would result in increased vaccine hesitancy. Similarly, according to Basch et al., (2021) most of the youths access misinformation from the social media such as the Tik Tok and is likely to cause COVID-19 vaccine hesitancy among the youths. The study found out that politicians’ sediments on COVID-19 vaccine was likely to trigger doubts on COVID-19 vaccine and therefore lead to increased vaccine hesitancy among the youths [26].

## Conclusion

Those who are ready to be vaccinated immediately stands at 42, 60% of them believed that their relatives and friends are ready to be vaccinated and 71% support for their community to have mass vaccination. As a result, 52% of youth are not ready to be vaccinated and are waiting to see what happens to those who have received the vaccine while 6% are totally unwilling. Therefore, total COVID 19 vaccine hesitancy stands at 58%.

In summary, respondents with lower level of education are willing to get vaccinated compared to counterparts with higher level of education.

The major determinants for their uptake of the vaccine were: concerns around safety and effectiveness of the COVID-19 vaccine, lack of right information, religious and cultural factors. Most youths felt that COVID-19 vaccine information was openly shared with social media being the main source of information for youths. However, social media was identified as a contributor of vaccine hesitancy among the youths as it was mentioned as a source of misinformation.

The study also revealed that majority of the youths support mass vaccination while 64.21% believe that once there are vaccinated with COVID-19 vaccine, other people in the community will be protected as well.

## Recommendations

It was recommended that the government and other stakeholders to design and implement a communication strategy on COVID vaccine to provide accurate information to the youth so that they can be able to acquire the right information. Also, the COVID-19 vaccine rollout should take into consideration the contextual gender disparities and should ensure that women are not left out in the vaccination. The Ministry of Health to enhance the vaccine trust among the population during the vaccination drives, and work closely with the protestant religious leaders more to reduce hesitancy among their flock.

## Data Availability

The data included in this manuscript were as collected from the respondents and analysed without alteration for any reason.

## Abbreviations

COVID-19: Coronavirus disease 2019
ETPB: Extended Theory of Planned Behaviour
NBCC: The National Business compact on Corona
SARS-Cov-2: Severe acute respiratory syndrome coronavirus 2
SPSS: Statistical Package for Social Sciences
TPB: Theory of Planned Behavior
WHO: World Health Organization

## Footnotes

1 The counties are further grouped based on urbanization percentages. The urbanization Percentages are obtained from Symbiocity Kenya 2015, https://symbiocity.org/wp-content/uploads/2019/10/1.-MAP-URBAN-DEVELOPMENT-FACTS.pdf . Counties with more than > 50% are categorized as urban, 20–49% are peri urban and less than 20% as rural counties

2
*Base outcome “I want to be among the first people to get the Vaccine*

3
*base outcome is “I want to be among the first people to get the Vaccine*

4
*base outcome “I want to be among the first people to get the Vaccine”*

5
*Base outcome “I want to be among the first people to get the Vaccine*
